# PathNarratives: Data annotation for pathological human-AI collaborative diagnosis

**DOI:** 10.3389/fmed.2022.1070072

**Published:** 2023-01-26

**Authors:** Heyu Zhang, Yan He, Xiaomin Wu, Peixiang Huang, Wenkang Qin, Fan Wang, Juxiang Ye, Xirui Huang, Yanfang Liao, Hang Chen, Limei Guo, Xueying Shi, Lin Luo

**Affiliations:** ^1^College of Engineering, Peking University, Beijing, China; ^2^Department of Pathology, Longgang Central Hospital of Shenzhen, Shenzhen, China; ^3^Department of Pathology, School of Basic Medical Science, Peking University Health Science Center, Peking University Third Hospital, Beijing, China

**Keywords:** pathology, human-AI collaboration, data annotation, multimodal data, colorectal cancer

## Abstract

Pathology is the gold standard of clinical diagnosis. Artificial intelligence (AI) in pathology becomes a new trend, but it is still not widely used due to the lack of necessary explanations for pathologists to understand the rationale. Clinic-compliant explanations besides the diagnostic decision of pathological images are essential for AI model training to provide diagnostic suggestions assisting pathologists practice. In this study, we propose a new annotation form, PathNarratives, that includes a hierarchical decision-to-reason data structure, a narrative annotation process, and a multimodal interactive annotation tool. Following PathNarratives, we recruited 8 pathologist annotators to build a colorectal pathological dataset, CR-PathNarratives, containing 174 whole-slide images (WSIs). We further experiment on the dataset with classification and captioning tasks to explore the clinical scenarios of human-AI-collaborative pathological diagnosis. The classification tasks show that fine-grain prediction enhances the overall classification accuracy from 79.56 to 85.26%. In Human-AI collaboration experience, the trust and confidence scores from 8 pathologists raised from 3.88 to 4.63 with providing more details. Results show that the classification and captioning tasks achieve better results with reason labels, provide explainable clues for doctors to understand and make the final decision and thus can support a better experience of human-AI collaboration in pathological diagnosis. In the future, we plan to optimize the tools for the annotation process, and expand the datasets with more WSIs and covering more pathological domains.

## 1. Introduction

Pathological diagnosis is the gold standard for most diseases, especially oncology, and is the cornerstone of clinical treatment (1). It studies the etiology, pathogenesis, and morphological changes of tissues and drives decisions about discovering, treating, and preventing diseases. With the development of deep learning and artificial intelligence (AI) technologies (2, 3), computational pathology has made significant strides in helping pathologists with auxiliary diagnostics and increasing their productivity in smart medicine applications such as classifying tumor subtypes (4–7), detecting cancerous regions (8), and segmenting lesion areas (9–11), especially for small and easily neglected lesion areas (12).

Artificial intelligence for pathology has stimulated a growing demand for high-quality pathological image datasets. Deep-learning-based computational pathology requires model training with numerous gigapixel whole-slide images (WSIs) scanned from H&E-stained specimens and annotated with diagnostic labels (13, 14). Due to its professionalism, pathological annotation usually relies on professional pathologists and is time-consuming and costly (15). The form and granularity of annotations imply the types of potential applications a dataset can support. For example, some large-scale datasets with WSI-level weak labels are used for weakly supervised classification tasks (16–18), while some datasets with region-level annotations can support more tasks of lesion segmentation with multiclassification types or even verbal explanations (19–21). Nevertheless, existing public datasets are not directly applicable for clinical use because most focus on the ground truth labels about what the diseases and lesions are, rather than why and how they are discovered and decided. As a result, the trained AI models can hardly provide enough diagnostic explanations for pathologists to understand the rationale.

There still exist challenges in collecting why and how annotations because pathologists’ diagnostic thinking logics are not well recorded and structured. Furthermore, the descriptions of a lesion’s decisive morphological characteristics are not consolidated due to the diverse captioning habits of pathologists. Most importantly, interactive annotation approaches must provide a flexible and systematic experience while avoiding additional workload for pathologist annotators (22).

In this study, we propose PathNarrative, a new annotation form that can collect both diagnostic labels and rich logical reasoning data for pathological AI to better collaborate with human pathologists. PathNarratives introduces an annotation protocol for pathologists to record both the decision-layer lesions and the reason-layer decisive features of diagnostic logic. It defines a hierarchical multimodal data structure to manage the decision-to-reason labels and their relations, a narrative annotation process, and an interactive tool to support annotators working in a flexible and multimodal way with clinical tags, voice, and pencil to not only mark the lesions but also point out the relative decisive features. Meanwhile, the underlying field-of-view (FOV) moving and pausing behaviors can be recorded simultaneously to together form the hierarchical annotation. Following the PathNarratives protocol, we recruited eight pathologist annotators and built a colorectal pathological dataset containing 174 WSIs with hierarchical decision-to-reason annotations. We further conduct experiments on the dataset with classification and captioning tasks to explore the clinical scenarios of human-AI collaboration in pathological diagnosis.

The major contributions of this study are as follows:

(1) A new annotation protocol, PathNarratives, that can obtain and manage clinical-compliant fine-grain multimodality labels, diagnostic thinking logic, and decision explanations. The hierarchical data structure involves decision-layer and reason-layer labels compliant with standard pathology clinical guides. A hierarchical terminology for the colorectal tumor is also proposed. Multimodality information labels are supported for flexible annotation.
(2) A comprehensive colorectal dataset of gigapixel WSIs with fine-grained annotations following PathNarratives was constructed. Each WSI involves the decision-to-reason hierarchical labels and the multimodality information.
(3) Exploration of the application scenarios of the PathNarratives colorectal dataset in diagnosis and experiments results show that finer labels improved performance in the classification and capitalization tasks. The explainable results supported doctors’ efforts to better understand and experience human-AI collaboration in pathological diagnosis.
  The rest of the study is arranged as follows. Section “2 Related study” the related study on datasets, narrative annotation, and relative AI applications. Section “3 Data annotation protocol” introduces the pathological data annotation protocol. Section “4 Dataset” presents the annotated colorectal dataset. Section 5 “Classification and captioning tasks on narratives-annotated dataset” shows the application scenarios and experiments on the dataset. Section “6 Conclusion” concludes and discusses future study.

## 2. Related study

### 2.1. Pathological datasets

Some pathology datasets are typically weakly labeled with simple metastatic disease circled at the WSI level and only applied to a single decision scenario (23–27). For example, CAMELYON16 (23) and CAMELYON17 (24) datasets have been widely used in research for automated detection and classification of breast cancer to enable automated evaluation of patient staging while reducing the subjectivity of the diagnosis. Similarly, the authors compiled TCIA (25) containing clinical information from epithelial ovarian cancer (EOC) and peritoneal serous papillary carcinoma (PSPC) to explore and develop methods for predicting the therapeutic effect of bevacizumab in patients with EOC and PSPC. The breast cancer dataset BreCaHAD (26) divides WSIs into six tissue classifications including mitosis, apoptosis, tumor nucleus, non-tumor nucleus, tubule, and non-tubule, to support multiclassification tasks. Another breast cancer dataset, BreaKHis (27), is designed for baseline classification of tumor benign-malignant and discrimination of subtype characteristic tissues. These dataset annotations only stay at the decision level of the metastatic region; the granularity is not detailed and persuasive enough.

Several pathological datasets aim to provide better clinical captioning to reflect pathology reports in computational pathology (19–21), including two categories. One was taken from existing digital resources, such as pathology textbooks and clinical and research journal article databases, which are typically represented by PathVQA (19) and ARCH (20). Such datasets are massive in volume but low in acquisition cost, poor in quality, and inconsistent in standards. These two datasets are often used for pre-training representational learning. During compilation, PathVQA also emphasizes templated and open-ended generation of visual question answers, compared to ARCH’s extracted image and image-related text pairs. Another type is obtained by picking patches from WSI, such as PatchGastricADC22 (28) and BCIDR (21). Among them, PatchGastricADC22 is derived from the actual clinical case diagnosis reports from the same hospital. Each instance has two magnifications, so the quality and resolution are consistent. Each WSI contains unorderly collected patches. Patches that belong to the same WSI have the same caption. Since there are only independent patches, there is no way to understand the mutual reasons for different patches in the doctor’s diagnosis. BCIDR allows more pathologists to participate in the annotation. The patches are extracted from eight typical regions and added captions, which makes their captions more focused on the detailed information at the cellular level. Thus, all of these datasets do not focus on region-level reasonable diagnostics. PathLAKE (22) proposes an annotation best practice that includes hierarchical case-level, region-level, and cell-level labels on breast cancer annotation but does not take the doctors’ diagnostic logic or the experience of multimodal inputs into consideration.

### 2.2. Narrative annotation model

Narrative annotation focuses on the description of the relationship between entities, and entity relationships are collected during the annotation phase. Attributes, relationships, and entities in the same image are often closely related (29–32). Localized Narratives (30) connect vision and language by artificially using mouse scribing to join action connections between entities and make the captioning in content more hierarchical. It asks annotators to describe an image with their voice while simultaneously hovering their mouse over the region they are describing. Using this mouse trajectory and voice inputs, the narrative dataset performs better in the caption task. Similarly, TReCS (31) exploits using detailed and reasonable language descriptions paired with mouse traces to generate images. More realistic images could be generated using descriptions and traces compared to those without traces. The interactions and relationships between objects contribute to a visual understanding of the main components of object-centric events (33). MITR (32) shows a framework to jointly model images, text, and human attention traces, which connects what to say with where to look by modeling human attention traces. The process of narrative annotation also contains helpful information in essence. By exploring the visual attention of doctors browsing and the process of scanning trajectories, Chakraborty et al. (34) found there are strongly correlated between the feature regions of algorithm tasks and lesions in the image to a certain extent, which reflects their diagnostic logic. The annotators draw the object’s bounding box with the mouse and add class labels through voice. Significant speed gains are achieved while maintaining high-quality annotations (35). In addition to manually adding entity relations during the annotation process, the models for video action recognition can also be considered partially auto-generating narrative relations of the entity bounding boxes (36–38).

### 2.3. Applications of AI in pathology

Medical classification and segmentation have also actively been explored (39–45). Gurcan et al. (39) reviewed pathological image analysis methods for computer-assisted diagnosis, including pretreatment, nucleus and gland segmentation, feature extraction, and classification. Veta et al. (40) discussed histological image analysis methods for breast cancer and conducted additional discussions on mitosis detection and proliferation assessment. Luo et al. (42) combined the characteristics of tumor cells and their surrounding organizational form environment to predict patient survival outcome information experimentally. HAG (43) was proposed to fuse multiresolution information and speed up prediction without reducing accuracy. Abu Haeyeh et al. (44) normalized the staining of RCC and used a weakly supervised multi-instance learning method. The results show that they can classify benign-malignant and determine tumor subtypes to support medical treatment management. Zhou et al. (45) chose TCGA, combing features at different magnifications, to achieve the classification and localization of colorectal tumors.

Pathological captioning tasks are being studied recently to automatically generate diagnostic texts based on patient medical images, assist inexperienced doctors, and reduce clinical errors (46). The typical representative is still PathVQA (19). PathVQA first reviews related research in medical radiology, such as VQA-Med (47) and VQA-RAD (48), and then explores the experiments of vision questions and answers tasks in pathology. The PathVQA automatically generates what, why, and other question-answer pairs to conduct the learning model by extracting pathological images and corresponding text information. In contrast to PathVQA, PatchGastricADC22 extracts patches from endoscopic biopsy specimens of gastric adenocarcinoma and trains an attention-based pipeline model to predict image features. The physician diagnostic logics of WSIs or lesion regions have not been extensively explored in the caption task at present.

## 3. Data annotation protocol

### 3.1. Overview

**FIGURE 1:**
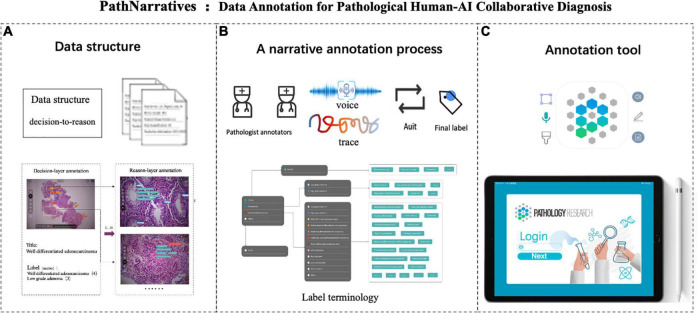
PathNarratives protocol including a hierarchical fine-grain data structure and a multimodal annotation process with an interactive annotation tool. **(A)** Decision-to-reason data structure. **(B)** Narrative annotation with label terminology. **(C)** Interactive annotation tool: ParVis.

We first analyzed the clinical routines of pathological diagnosis to formulate the annotation data structure and the protocol of PathNarratives, as shown in Figure 1. To be specific, we consulted the WHO pathological clinical guideline (49), analyzed the pathology report templates from the pathology departments of two top-tier hospitals, and observed two pathologists for their diagnosis browsing and thinking practices with permission (P4 and P9 in Table 1). The goal was to explore how pathological decisions are made, explained, and concluded into reports, and what granularity of interpretable annotations can be collected in a natural process.

**TABLE 1:** Basic information of participating pathologists.

Pathologist	Years-of-working	Subspecialty
P1	More than 15 years	Histopathology
P2	3–5 years	Histopathology
P3	3–5 years	Histopathology
P4	5–10 years	Digestive
P5	10–15 years	Digestive
P6	10–15 years	Histopathology
P7	3–5 years	Histopathology
P8	More than 15 years	Histopathology
P9	3–5 years	Digestive

We then defined the PathNarratives protocol, which includes a hierarchical decision-to-reason data structure, a multimodal annotating process, and an interactive annotation tool. It allows annotators to work in a flexible and multimodal way to mark and circle lesion areas, look for typical characteristics and outline them, and describe the basis of judgment, by using clinical tags, voice, pencil lining, and FOV moving. Following this, the collected data can cover the types of diagnostic disease and lesion, the decisive morphological features, and the corresponding pathologists’ logical narrations and viewing behaviors.

### 3.2. Data structure

#### Decision-to-reason annotation

Concluding a pathological diagnosis report involves two layers of information. The decision-layer information is about the slide-wise diagnostics (one report may involve several slides of the patient) and descriptions of lesion regions that appear explicitly in the pathology report. In contrast, the reason-layer information demonstrates the underlying typical features and reasons that pathologists use to judge the lesion and diagnose it. Although the reason-layer information is essential to explain the rationale, it is usually implicit in pathologists’ knowledge systems and does not show in the report. Only when pathologists discuss with other doctors will they refer to both the decision-layer and reason-layer information of the diagnosis, using multimodal ways such as texts, voice, screenshots, and mouse/pencil moving.

Besides the two layers of information, we discovered that doctors’ behaviors such as browsing, view zooming-in/out, view shifting, view pausing, and mouse/pencil hovering represent their attention focus and thinking logic during the pathological diagnosis process. Such behavior data also provide informative inputs for AI learning and, therefore, are also considered in our data structure.

**FIGURE 2:**
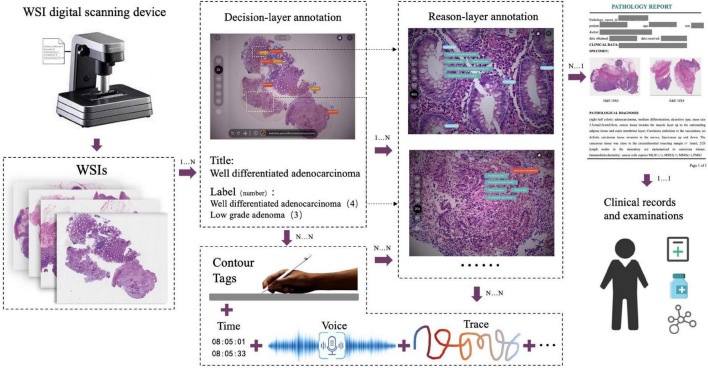
The decision-to-reason multimodality data structure of PathNarratives. The decision-layer represents the labels around WSIs and lesion regions, where each WSI corresponds to multiple lesion regions. The reason-layer represents the corresponding multiple features marked and described to judge the lesion regions.

The decision-to-reason data structure to manage the hierarchical multimodal annotation is shown in Figure 2. The decision-layer represents the labels around WSIs and lesion regions, where each WSI can involve multiple lesion regions (one-to-many mapping, shown as 1…N in Figure 2). The reason-layer is related to the corresponding multiple features labeled with descriptions to explain the rationale behind judging each lesion decision (one-to-many mapping, shown as 1…N). Multimodal annotations are supported as clinical tags, free texts, voice, and pencil/mouse moving traces of the doctor’s annotating behaviors, which are timestamp synchronized and associated with both the layers of data (many-to-many mapping, shown as N…N). Multiple annotations together form one comprehensive pathology report (many-to-many mapping, shown as N…N).

#### Unified terminology

We also considered the need for unified terminology of the two layers of labels in the data structure design, where the colorectal tumor is chosen in this study. During the pathological shadowing, we found that if we allowed two pathologists to input free-text reasoning labels, their expressions could vary severely even when they agreed on the tumor types and reasons for the same lesion of a colorectal WSI. For example, pathologist 4 (P4 in Table 1) described the features as a “gland fused with a sieve,” while Pathologist 9 (P9 in Table 1) described the same one as a “sieve hole.” Further interviews with the two doctors proved that they meant the same thing, though their textual expressions looked quite different. The variability of labels affects not only the performance of the AI model but also the normalization of data, and therefore, unified terminology is necessary.

We analyzed pathological books, published specifications, and pathology report templates from hospitals and consulted senior pathologists (P1 and P9 in Table 1 with more than 15 years of diagnostic experience) to build the decision-to-reason unified terminology, as shown in Table 2 (refer Supplementary material for the full version). We first referred to the 2019 WHO Blue Book (World Health Organization) (50), which defines the classification of digestive system tumors and borrowed the colorectal classification terms to form the overall classifications as “normal, adenocarcinoma and adenoma.” Besides the WHO Blue Book, comprehensive pathology report templates from two top-tier hospitals in China are also considered to further define the finer classification of the decision-layer label, e.g., “Adenocarcinoma” in the classification is categorized into subtypes such as “Poorly differentiated adenocarcinoma” and “Moderately differentiated adenocarcinoma.” In addition, some terms that frequently occur in pathology reports describing features of lesions, such as “Tumor invasion,” “Tumor budding,” “vascular invasion,” and “nerve invasion,” are also set as decision-layer labels to better accommodate pathologists’ habits and clinical needs.

**TABLE 2:** Label terminology partial (in total, there are 3 classification labels, 12 subtypes, and 77 reason-layer labels).

Classification label	Decision-layer subtype label	Reason-layer label
Adenocarcinoma	Poorly differentiated adenocarcinoma	Irregular arrangement of glands Mucinous differentiation Vacuolated nuclei …
	Moderately differentiated adenocarcinoma	
	…	
	Tumor invasion	Infiltration of single or several tumor cells Invasion into the muscularis mucosae …
	Tumor budding …	Tumor budding (grade 1) …
Adenoma	Low-grade adenoma	Low-grade intraepithelial neoplasia Glands lack mature differentiation …
	High-grade adenoma	…
Normal	Normal	Fatty tissue Smooth muscle Lymphatic vessel …

Specifically, adenocarcinoma is mapped to 9 decision-layer subtypes and 34 reason-layer labels; adenoma is mapped to 2 decision-layer subtypes and 25 reason-layer labels; normal is mapped to 1 subtype and 18 reason-layer labels.

Reason-layer label terminology was designed under the decision-layer labels. As the WHO book and pathology reports do not involve detailed reasoning information, we invited the senior pathologists to summarize the main features into the reason-layer annotation description from textbooks (51) with consideration of the decision labels and pathology reports. As shown in Table 2, “Poorly differentiated adenocarcinoma” in the decision-layer is further associated with detailed reason-layer labels describing diagnostic features such as “Irregular arrangements of glands” and “Mucinous differentiation.” Specifically, the decision-layer labels under the “Normal” category are used to describe normal colorectal elements such as “Fatty tissue,” “smooth muscle,” and “Lymphatic vessel.” The terminology terms are ordered from histomorphology to cell morphology for pathologists’ convenience in browsing and selecting from it.

### 3.3. Annotation process and tool

**FIGURE 3:**
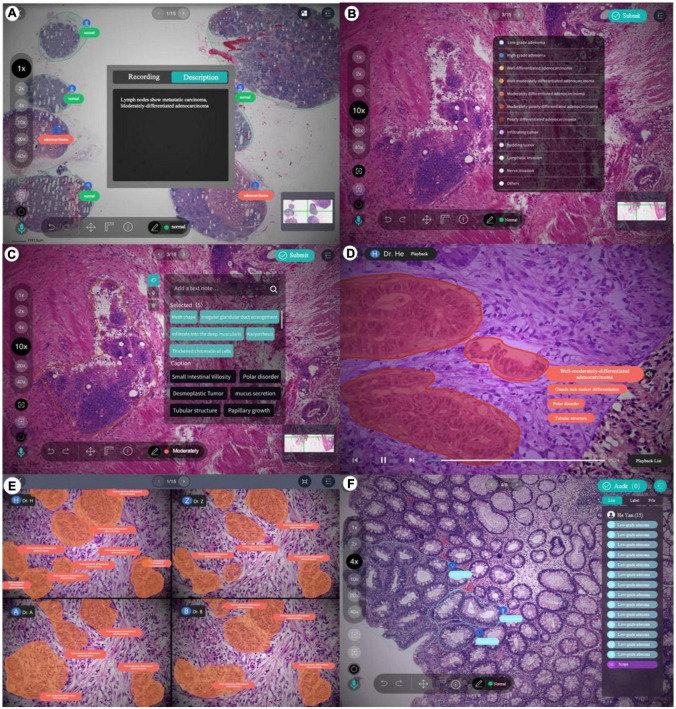
Decision-to-reason annotation functions of ParVis. **(A)** Label module: Lesion area circled on WSI by an annotator in the coarse-grain annotation phase, with preliminary diagnosis descriptions. **(B)** Label module: Decision-layer subtype labels as different colors of pencils in the fine-grain annotation phase. **(C)** Label module: Reason-layer features circled and labeled by clicking on the reason-layer terminology tags or recording voice explanations, in the fine-grain annotation phase. **(D)** Playback module to replay the annotation events on the WSI image which is structural and can be searched and analyzed. **(E)** Comparison module to view and compare different doctors’ annotations. **(F)** Audit module for senior doctors to review and correct previous annotations.

The PathNarratives annotation process includes a coarse-grain phase and a fine-grain phase that follow the decision-to-reason labeling structure. The design of the two phases is to accommodate the different clinical application needs such that in the coarse-grain annotation phase, an annotator browses a WSI and circles large lesion areas to tag with the classification labels and then makes a preliminary slide-wise diagnosis description, as shown in Figure 3A. This annotation phase can be completed quickly by doctors and an overview diagnosis can be provided. Then, in the fine-grain annotation phase, an annotator needs to circle the finer subtype decisions of lesions with typical features as completely as possible and explain the decisive reasons. They can use a decision-layer subtype label pencil to circle the typical lesion features, and then either attach reason tags or record voice explanations to explain the diagnostics. The decision and reason labels can be directly picked from the predefined label terminology, as shown in Figures 3B, C. The fine-grain phase is more sophisticated and requires more time and labor. Images and annotations can be replayed, compared, and audited afterward, as shown in Figures 3D–F, respectively.

The above annotation process is carried out using our self-developed software ParVis for the convenience of pathologist annotators, auditors, and project managers cooperating on an annotation project. The software comprises a mobile client for doctors’ daily annotation/audit and a web server for annotation project management. Administrators create projects, upload pathology images, set roles and access rights, and manage terminologies through the web server. Pathologists use the mobile client to join projects, submit annotations, review them, and audit the results.

According to the annotation process, ParVis has four major functions: label, playback, review, and audit. On the label module interface in Figures 3A–C, a pathology annotator can start labeling a WSI for coarse annotation of the slide-level diagnosis description and use different colors of classification pencils to mark lesion area contours as in Figure 3A. For further fine-grain annotation, ParVis provides different colors of subtype pencils for the annotator to circle the contours of typical lesion features as in Figure 3B, and the icons of “mic” or “tag” can be clicked to describe the features with voice or text to generate the reason-layer labels in Figure 3C. In addition to colors, the pencil tool supports flexible shapes for marking lesion areas, such as “curve,” “rectangle,” or “brush.” ParVis also provides a “ruler” to measure the area size according to the needs of pathological reports. The fundamental functions such as magnification rate, eagle view, screenshot, location, and metadata view are also provided as basic functions.

ParVis forms the structural multimodal annotation data for further analysis, playback, review, and audit. It also periodically records the timestamps of browsing and moving behavior events during labeling (with doctors’ prior permission) for further synchronization. The behavioral tracking includes events such as “FOV center change,” “voice recording,” “magnification,” “pencil switching,” “undo,” and “delete” over time during the doctor labeling process. These data can support application modules of playback (to replay the annotation process), comparison (for medical students to review and learn from multiple experts or teachers to examine multiple Students’ work simultaneously), and audit (for auditors to review and refine the annotations), as shown in Figures 3D–F. Most importantly, the synchronized events such as magnification and focus center shifting implicitly recorded can be used to analyze physician behaviors. For example, visualizing the FOV center trajectory shows the length of stay is positively correlated with the difficulty of the lesion area, which is consistent with the conclusion in Wang and Schmid (37). Behavioral data indicate the logical thinking of doctors and their attention to assist the interpretability of AI.

The audit is an essential step for the annotation process to ensure data quality and consistency, which needs to be conducted by senior pathologists. The ParVis audit module is designed following the general practice of the pathology department. A senior pathologist clicks the Audit button and selects the items marked by primary pathologists and checks for missing or wrong annotations. If there is a problem, they need to revise, add, or delete the labels to finalize the submission. We use Kappa, Dice, and BLEU to evaluate the consistency of different levels of annotations in section “4.1 Data source and overall statistics.”

During the annotation practice, we kept optimizing the process according to observed issues. One important issue is the cost of fine-grain annotation to label all the reasoning tags, which is tedious and expensive for pathologists even though it provides more details and explanations. Since many adjacent glands or lesions share similar characteristics, we added a “Bundle pencil” tool to support annotators to circle adjacent lesion regions of similar reasoning tags, so that a pathologist can simply apply a one-off description to all the lesions and features within the bundling circle. This setting saves annotation time to a considerable degree in practice.

## 4. Dataset

### 4.1. Data source and overall statistics

Based on the PathNarratives protocol, we recruited eight pathologist annotators (P1–P8 in Table 1) to build a colorectal tumor dataset, CR-PathNarratives, which includes 174 annotated colorectal WSIs with a length of 8,000–90,000 pixels and width 6,000–60,000 pixels, all with the decision-to-reason and multimodal data structure.

We selected colorectal cancer because it is characterized by high incidence and mortality. Colorectal cancer has become the second leading cause of cancer death worldwide, with 930,000 deaths in 2020. In 2020, the new incidence rate of colorectal cancer in China was 12.2% and the fatality rate of colorectal cancer was 9.5% (52). In addition, colorectal tissue sections present explicit morphological variance and cover wide categories of tumor types with well-established pathological diagnostic guidelines and standards for database design and practice.

The WSIs were obtained from one first author’s cooperative hospital with approval. The chief pathologist selected 891 H&E-stained slides from 300 patients and randomly sampled 300 pieces to scan into WSIs at 20X objective magnification. At present, the collection of annotated data containing 174 WSIs has been completed.

We conducted the basic statistics of CR-PathNarratives on the distributions of classification types, decision-layer subtype labels, reason-layer labels, labeled areas, and diagnostic captions composed with reasoning labels. The dataset covers all three class types: adenocarcinoma, adenoma, and normal. The detailed categories and numbers are shown in Table 3.

**TABLE 3:** Subtype distribution and data scale table.

Classification	Decision subtype	Number of WSIs in the subtype	Total
Adenocarcinoma	Well differentiated	20	108*
	Poorly differentiated	23	
	Moderately differentiated	26	
	Well-moderately differentiated	16	
	Moderately-poorly differentiated	23	
Adenoma	High-grade adenoma	25	38*
	Low-grade adenoma	13	
Normal		45	45

*Indicates that 17 lesion slides contain both adenocarcinoma and adenoma.

**FIGURE 4:**
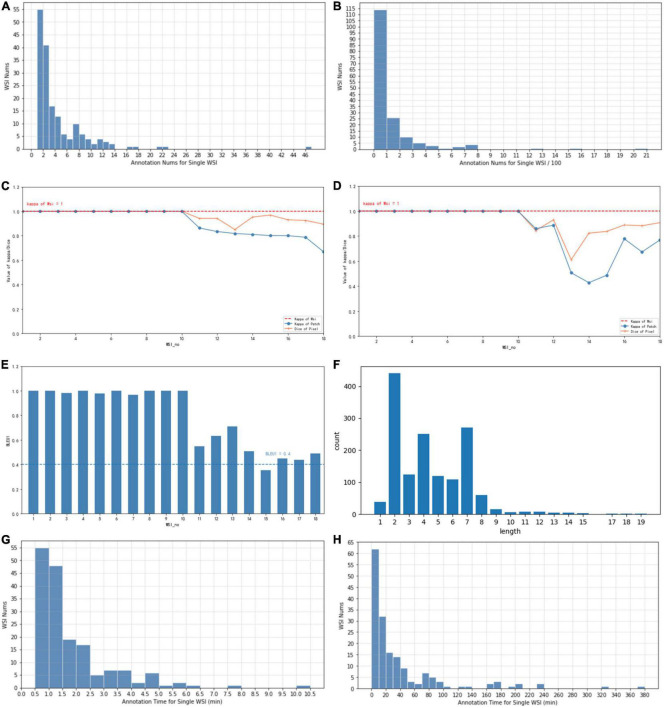
**(A)** The typical number of annotations contained in a single WSI of coarse-grain phase. **(B)** The typical number of annotations contained in a single WSI of fine-grain phase. **(C)** Consistency of different annotators with Kappa and Dice values of coarse-grain annotation data. **(D)** Consistency of different annotators with Kappa and Dice values of fine-grain annotation data. **(E)** Consistency of different annotators with BLEU1 of description data. **(F)** Distribution of caption length and number. **(G)** The time spent on a single WSI annotation of the coarse-grain phase. **(H)** The time spent on a single WSI annotation of the fine-grain phase.

Each WSI contains a simple overall caption, several decision-layer labels, and tens to hundreds of reason-layer labels. In total, in 174 WSIs, 108 contain adenocarcinoma areas ranging from well differentiated to poorly differentiated, 38 contain adenoma areas, 17 contain both adenoma and adenocarcinoma, and 45 are normal slides with only normal areas labeled. There are in total 11 types of decision-layer labels and 75 reason-layer labels, including free-text tags. For the whole dataset, there are 23,532 regions manually circled, and some are grouped as 539 bundles in total (a bundle consists of multiple or single regions sharing the same features and captions, which can effectively reduce the labeling efforts, as mentioned in section “3.3 Annotation process and tool”). In total, there are 878 different kinds of captions associated with all the labeled regions, and each caption comprises 4.4 label terms on average (max = 19 and min = 1), as shown in Figure 4F.

Whole-slide image-wise statistics show that on average, a WSI contains 3.1 labeled bundles (max = 41 and min = 1) that reflect 135.2 regions. For further AI algorithm computation, each WSI scanned at 20 × magnification was cut into patches of 256*256 pixels. In statistics, the averaged labeled regions contain 76 patches (the diversity ranged from max = 2,477 to min = 1). On average, one WSI is associated with 8.93 different kinds of captions (max = 40 and min = 1) and involves 12.03 reason label terms (max = 42 and min = 1).

We also investigated the texts and captions frequently used in annotation statistics. The most commonly used label terms are “Stratified or pseudostratified arrangement of nuclei” (7.21%), “Rod-shaped nuclei” (7.02%), “Increased layers of epithelial cells” (5.78%), and “Chromatin condensation of cells” (5.50%). For reason-layer labels, the most commonly used captions are “Mitosis visible, mucous differentiated, vacuolated nuclei,” “Markedly reduced cytoplasm, stratified or pseudostratified arrangement of nuclei, increased layers of epithelial cells, rod-shaped nuclei, oval nucleus,” and “Cribriform.”

We also evaluated the consistency of doctors’ annotations for the quality of the datasets. For 10% of the annotated samples (18 WSIs), we asked a senior doctor P4 to review and label the same WSIs annotated by a senior doctor P5 and a junior doctor P2. Three levels of annotation consistency are analyzed as shown in Figure 4 (WSI number sorted by their consistency value for illustration): consistency of WSI classification in (c), (d), consistency of lesion regions for coarse-grain classification labels in (c) vs. fine-grain subtype labels in (d), and consistency of reason descriptions of lesion features in (e), measured with the Kappa, Dice, and BLEU values, respectively. For the consistency of WSI classification, the types decided by both doctors are all the same for the 18 WSIs, which achieves an overall Kappa = 1. For the consistency of lesion regions, the patch-level classification labels and decision subtype labels achieve an average Kappa of 0.91 (max = 1, min = 0.66) and 0.85 (max = 1, min = 0.42), respectively, while the pixel-level consistency of the same-label lesion area achieves Dice values of 0.96 (max = 1, min = 0.85) and 0.92 (max = 1, min = 0.61) for classification and subtype labels, respectively. Both the patch-level Kappa value and the Dice value are with an average beyond 0.85, and the variance among different WSI is considered due to the difficulty levels of different cases. For consistency of reason descriptions represented by lesion caption, the BLEU1 value is mostly beyond 0.4 with an average of 0.78, as shown in Figure 4E.

Annotation auditing is widely used in clinical practice. When inconsistency occurs, the primary annotator needs to double check, and if there is still a dissenting opinion, the senior and primary annotators need to communicate with each other to achieve a consensus.

### 4.2. Decision-to-reason annotation

**FIGURE 5:**
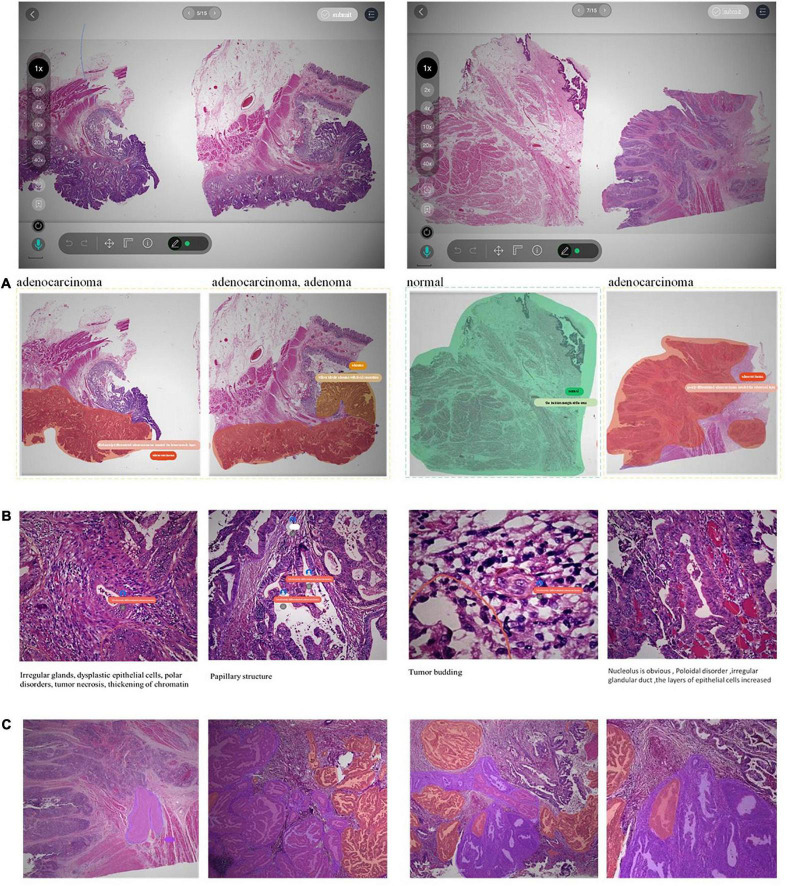
Examples of decision-to-reason annotation data. **(A)** The annotation of coarse-grain phase. **(B)** The annotation of the fine-grain phase. **(C)** The annotation of fine-grain phase with bundle label.

The two layers of decision-to-reason data are shown as examples in Figure 5. A doctor would rather look at the typical reason-layer features first to quickly conclude the diagnosis and lesion areas, and then spend much more time explaining with subtype details, typical features, and reasons. For example, the doctor looked at the lesions on a WSI that present visual features such as “Cribriform,” “nucleus stratified or pseudostratified arrangement,” and “polar disorder” and then quickly marked the whole WSI as “moderately differentiated adenocarcinoma” and circled two adenocarcinoma regions and one adenoma region. Then they refined to circle more reasoning feature regions and select the detailed reason-layer labels for fine-grain annotation.

Artificial intelligence training requires the annotations to be as complete as possible. Coarse-grain labeling is simpler and costs less time because doctors roughly scan the lesions and add labels to the low-resolution WSI, which takes only tens of seconds. In contrast, though it contributes necessarily detailed reasoning information, fine-grain labeling inevitably takes a longer time in marking all the circles and label terms. Experiments show the time of coarse-grain labeling per WSI is on average 1.7’ as shown in Figure 4G, ranging from 0.29’ to 2.97’, while the time spent for fine-grain labeling is on average 46.17’, ranging from 14.69’ to 98.83’ as shown in Figure 4H, which is 20+ times of that for coarse-grain one.

Fortunately, by applying the proposed “Bundle pencil” to group similar small lesion regions for the one-off application of the same labels as shown in Figure 5C, the fine-grain annotation time can be significantly reduced down to 1/6–1/2 of the original one. We also found it uses more time for the doctor to label adenomas than to label adenocarcinomas because the lesion areas of adenocarcinomas are often tangled and cannot be labeled separately. It also took much time to zoom back and forth to inspect a large lesion area and label all the typical details at different views. Based on this finding, we proposed the following methods to further reduce the burden of doctors. (1) Use the “Bundle pencil” to circle lesion areas with similar features and (2) Future exploration of AI technologies to provide automatic hints for circling and labeling.

Taking the WSI shown in Figure 5 as an example, the WSI was marked with 12 adenocarcinoma areas, 9 adenoma areas, and an overall cost of 1’12” for coarse-grain labeling, and the adenoma was described with the text “Low-grade intraepithelial neoplasia.” During fine-grain annotation, the doctor marked 83 well-differentiated adenocarcinomas, 45 low-grade adenomas, and added 8 bundle tags, which overall cost 7’42”. In another example case, annotating a WSI takes a doctor 12” to circle 3 lesion regions with classification labels, while annotating the fine-grain 488 typical features with diagnostic reasons take up to 31’24” for no-bundle-circle annotation vs. about half of it for bundle-circle annotation. In contrast, by simply applying the “Bundle pencil” to group similar small lesion regions and one-off label them, the annotation time is significantly reduced to 14’52”, which is less than half of the previous time.

### 4.3. Multimodal data

**FIGURE 6:**
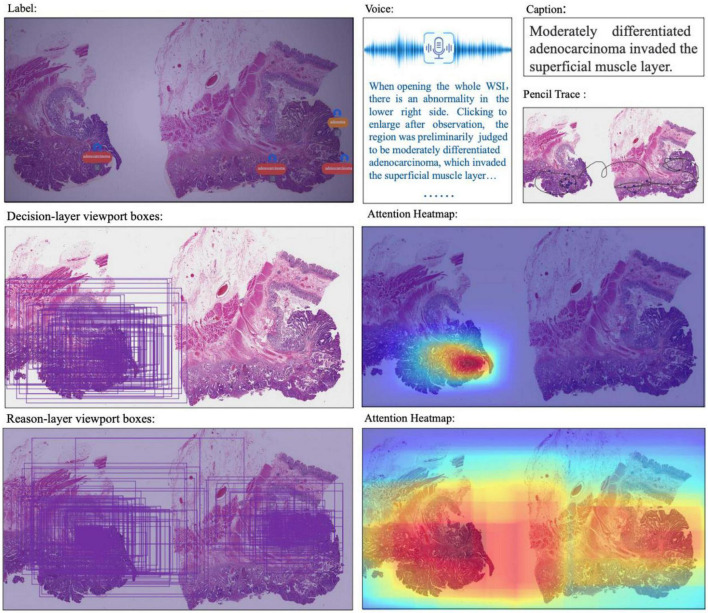
Multimodal information in the dataset: WSI, annotated information, voice, caption, ratio of decision-labeled and center point trace of the viewport, ratio of detailed labeled, and center point trace of viewport and corresponding generated heat map.

Besides decision-to-reason data, CR-PathNarratives also covers multimodal annotation data. Each WSI in the PathNarratives dataset has visual information on the image feature regions and language information of the physician’s annotations described in section “4.2 Decision-to-reason annotation.” On the contrary, the PathNarratives dataset also contains voice information and behavioral trajectory information, according to doctors’ preferences. From the example shown in Figure 6, we found that voice information mainly consists of the following two types of purposes: explaining diagnosis by thinking or labeling *via* voice. We observed that after his annotation, the doctor turns on the voice record button and tries to elaborate on his observation for teaching purposes, e.g., “Open the whole WSI and find that the right side is somewhat abnormal. Click to enlarge and observe to confirm the adenocarcinoma. On the left side, there are irregular glandular and tubular arrangements and invasion of the muscle layer.” Junior physicians can replay and listen to learn the voice-input recordings about WSI colorectal diagnostic methods, which shares similarity to the AI learning process. The voice-transcribed text labels contain richer information among the marked areas and complement the textual label terms. However, our experiment does not involve the special natural language processing needs for pathological text recognition, which is an in-depth research area. Instead, we only recruited human medical students to perform that transcribing tasks.

The behavior-tracking data of doctors are stored in a structured time-series record of labeled behaviors such as time stamps, visual field centers, magnifications, labeling tools, toggle label colors, markers, coordinates, deletions, and modifications during their labeling process. When a doctor labeled a WSI, we continuously recorded his FOV window changing, visual scan path, and resolution zoom in and out information. We visualized the doctor’s attention distribution of diagnosis by aggregating the pixels of the doctor’s viewport boxes, combining them with the center points of the viewport boxes, checking the time, zooming into incorporating scan path, and plotting a behavioral trajectory heatmap as shown in Figure 4, 5. The attention heatmaps echo the areas that the doctors observed the most with higher heat scores. In comparison, tracks of junior physicians demonstrate more back-and-forth browsing and reluctance than those of the senior pathologists who are experienced to make diagnoses rapidly.

## 5. Classification and captioning tasks on the narratives-annotated dataset

**FIGURE 7:**
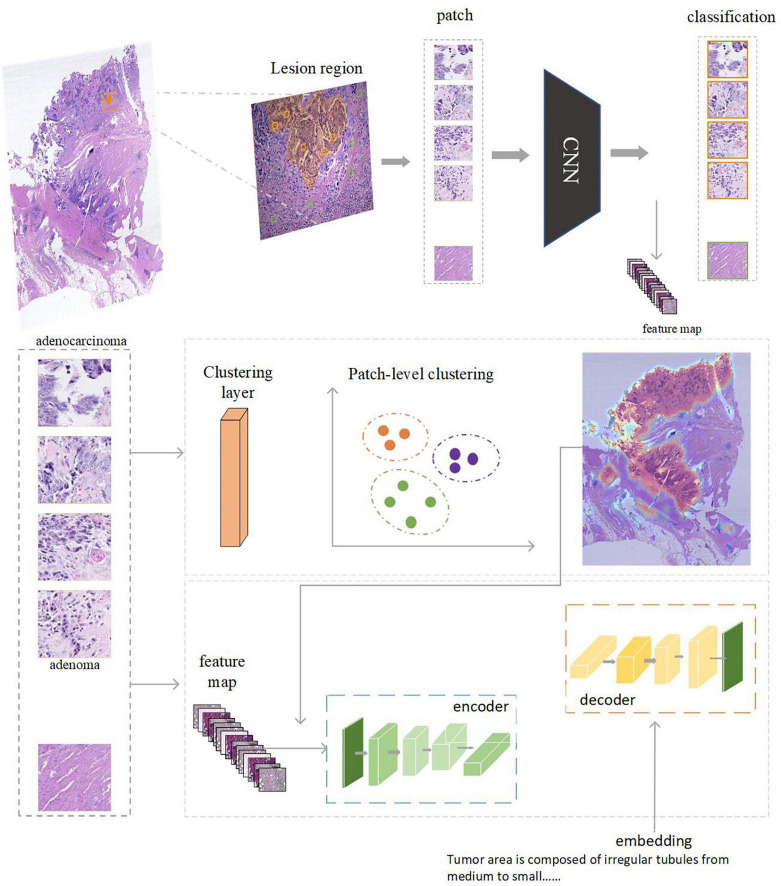
The AI model consists of two parts: feature extraction and classification using CNN. Captioning with transformers. Clustering can be performed based on the regions annotated by doctors. After each patch is classified and clustered, the captioning of clustered areas can be performed.

To investigate the potential clinical applications that the CR-PathNarratives dataset can support, we selected a classification task and a captioning task and trained the baseline AI models. We also conducted an evaluation of Human-AI collaboration experience to explore the doctor subjects’ trust and acceptance when being provided with comprehensive decision-to-reason suggestions by AI models. The experimental baseline AI model is shown in Figure 7.

### Task 1: Classification of coarse-grain and fine-grain labeling data

#### Task definition

Given a WSI with coarse-grain classification labels vs. fine-grain subtype labels defined in Table 2, the goal is to compare their performances of classification (normal, adenocarcinoma, and adenoma) to explore the impact on different levels of labeling details. For ideal clinical use, false negatives should be avoided, which means a WSI containing adenocarcinoma should not be misjudged as an adenoma or benign case.

#### Methods

Each WSI is assigned a universal ID. We used the OpenSlide tool (53) to extract patches of 256*256 pixels from WSIs at 20 × magnification. Macenko stain normalization (54) is used for pre-processing to ensure uniform WSI quality. The OTSU algorithm (55) is used to separate foreground and background, ensuring that all valid patches come from the foreground tissues.

The training and test sets are first divided into the WSI grade to avoid patches from the same patient being included in both sets. The total cropped tissue patches for training were counted, where a patch is regarded as a labeling type if its central pixel falls into the region labeled with that type. For each WSI, the patches with one labeling type were randomly sampled according to the overall ratio of the type in the dataset. Normal patches are guaranteed to come from normal WSIs rather than normal areas of tumor slides. The test set is composed of four WSIs with two adenoma and two adenocarcinoma ones, cropped as patches with stride 256 in X and Y directions without overlap area. The numbers of sampled patches are shown in Table 4.

**TABLE 4:** The number of sampled patches for the training set and test set for the classification task.

	Normal	Adenocarcinoma	Adenoma
Training set with coarse-grain classification labels	133,312	133,321	133,286
Training set with fine-grain subtype labels	133,312	133,322	133,252
Test set	15,244	4,197	10,603

ResNet-50 (49) is used for patch feature extraction and classification in our experiments. The same setting (batch size = 128, classes_num = 3) is used to perform the classification of the tumor, carcinoma, and normal cases. We used Adam to optimize the model with an initial learning rate of zero and âtaken from the set of (0.9, 0.999). After five warm-up epochs, the learning rate reached 0.001. Then, CosineAnnealingLR was chosen as the learning rate decay strategy, and after 25 epochs, it decayed to zero. Experiments were run with PyTorch on a machine with a V100 graphics card.

#### Evaluation

We evaluated the performance with precision, recall, and accuracy indicators. Precision is to measure how many of the positive predictions are positive. Recall tells how many positive cases in the test set are predicted correctly. Accuracy reflects the overall ratio of correct predictions (adenoma, adenocarcinoma, and normal).

#### Results

Table 5 shows that fine-grain prediction enhances the overall classification accuracy from 79.56 to 85.26%, with a +5.7% improvement compared with the coarse-grain one. In specific, for normal class, the recall measure of fine-grain prediction outperforms that of the coarse-grain prediction up to +11.29%, from 67.83 to 79.12%. For adenocarcinoma, coarse-grain prediction results in a small false negative, reaching the recall of 89.46%, while fine-grain one further improves it up to 90.71%. The fine-grain recall measure of adenoma is also good at 93.70%, though is −3.76% inferior to the coarse-grain one, and one possible reason is that some tumor stroma characteristics are difficult to identify. In conclusion, experimental results show that fine-grain annotations can achieve an overall good performance of classification and indicate more details of the present lesions.

**TABLE 5:** Confusion matrix of prediction results for models trained with coarse-grain classification labels vs. fine-grain decision-layer subtype labels.

		Prediction			
	Ground truth	Normal	Adenoma	Adenocarcinoma	#Recall
Coarse-grain class data	Normal	5317	483	2039	67.83%
	Adenoma	28	1687	16	97.46%
	Adenocarcinoma	624	26	5517	89.46%
	**#Precision**	89.07%	76.82%	72.86%	**79.56%**
Fine-grain subtype data	Normal	6202	297	1340	79.12%
	Adenoma	69	1622	40	93.70%
	Adenocarcinoma	565	8	5594	90.71%
	**#Precision**	90.37%	84.17%	80.21%	**85.26%**

Recall and precision numbers are calculated, and the two boxed numbers represent the overall accuracies of the two models, respectively.

### Task 2: Caption generation for explaining diagnosis rationale

#### Task definition

Besides classification, we further verify the effectiveness of reason-layer data in explaining details for the classification rationale in order to support clinical scenarios of pathologists-AI collaboration. We designed a captioning experiment to compare the descriptions annotated by the doctor with the region captions generated by the AI model. We also conducted a subjective evaluation for doctors to review the captions generated.

#### Methods

The captioning model consists of a Resnet-18 (49) backbone network and a transformer (56). Between the two modules, we inserted a clustering filter module to aggregate patches belonging to the same lesion area into ac luster. The model accepts random patches as input, extracts features *via* the backbone network, and predicts the classification type (normal, adenoma, and adenocarcinoma) of the patch. The clustering filter will then aggregate adjacent abnormal patches into clusters representing the lesion areas. Each cluster contains several patches, which are regarded as a bag of unordered patches. All the patch features in this bag are fed into the transformer to generate the corresponding caption.

All the labeled lesion areas were divided into several patches with corresponding captions for training purposes. For tokenization purposes, patches in each caption bag are sampled to a fixed number. Specifically in the experiment, we set the number of patches per caption as up to 64. During the testing phase, the DBSCAN (57) clustering filter was used after the backbone was completed. Each cluster generated by the clustering filter was into the transformer to generate the caption. We used a Tesla V100 graphics card for training with batch size = 4; AdamW was used as the optimizer with a learning rate of 1e-5. In the test stage, we sampled up to 256 patches per cluster for caption prediction.

#### Evaluation

The bilingual evaluation understudy (BLEU) (58) score was adopted for quantitative region-level algorithm evaluation. BLEU value is used to measure the similarity between a set of machine-generated translation sentences and a set of human-translated sentences. A higher score reflects a better agreement between the caption produced by the model and the ground-truth description by the annotator.


(1) $$b\,l\,e\,u_{n}=\frac{\sum_{c\in c\,a\,n\,d\,i\,d\,a\,t\,e\,s}\sum_{n-g\,r\,a\,m\in c}C\,o\,u\,n\,t_{c\,l\,i\,p}\,\left(n-g\,r\,a\,m\right)}{\sum_{c^{\prime }\in c\,a\,n\,d\,i\,d\,a\,t\,e\,s}\sum_{n-g\,r\,a\,m^{\prime }\in c^{\prime }}C\,o\,u\,n\,t_{c\,l\,i\,p}\,\left(n-g\,r\,a\,m^{\prime }\right)}$$

#### Results

We used four grades of BLEU values B1, B2, B3, and B4 to quantify the captioning results. Experiments showed that the model achieved B1 = 0.56, B2 = 0.49, B3 = 0.44, and B4 = 0.36, for which the predicted captions demonstrated good similarity to the ground truth descriptions (BLEU around or higher than 0.4). Some examples are shown in Table 6 for better illustration.

**TABLE 6:** Partial caption prediction result.

BLEU4	Predicted caption	Original caption
0	The cytoplasm was markedly reduced, karyorrhexis, thickened chromatin, screen mesh. Moderately differentiated adenocarcinoma.	Nuclei remain polar, nucleus stratified or pseudostratified arrangement, tubular structure, increased epithelial cell hierarchy, low grade intraepithelial neoplasia. Low grade adenomas.
0.3	Irregular glandular duct arrangement, cribriform structure, the nucleus of tumor cells are round, nucleoli were more prominent, necrosis. Moderately differentiated adenocarcinoma.	Some tumor cells with round nucleus, nucleoli were more prominent, some cribriform arrangement, some papillary arrangement, necrosis, some tumor cells rod-shaped, stratified arrangement. Moderately differentiated adenocarcinoma.
0.45	Irregular glandular duct arrangement. Moderately differentiated adenocarcinoma.	Infiltration into the submucosa. Moderately differentiated adenocarcinoma.
0.5	Nuclei rod-shaped, nucleus stratified or pseudostratified arrangement, tubular structure. Low grade adenomas.	Nuclei rod-shaped, nucleus stratified, tubular. Low grade adenomas.
0.99	Nuclei rod-shaped, nucleus stratified or pseudostratified arrangement, tubular structure. Low grade adenomas.	Nuclei rod-shaped, nucleus stratified or pseudostratified arrangement, tubular structure. Low grade adenomas.

### Task 3: Human-AI collaboration experience

**FIGURE 8:**
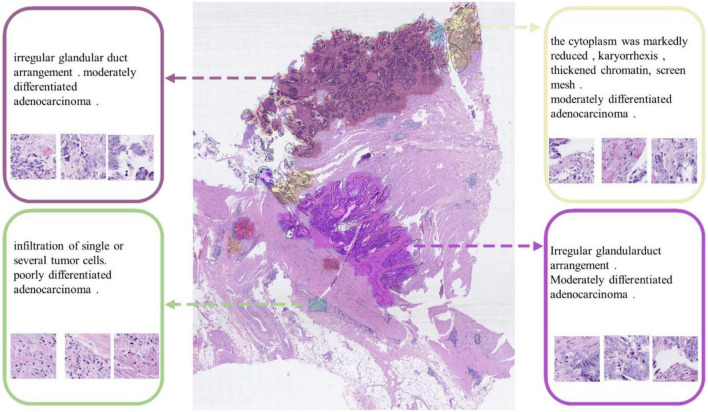
Captions generated for each clustered area. These captions are used to describe the lesion detail features of the clustered area.

We also engaged physicians in qualitative evaluation of the captions at the cluster level. For a certain WSI for testing in Task 1, after completing the ResNet-based classification, we used DBSCAN to cluster the patches and visualize the clustering result as shown in Figure 8. All lesion regions are clustered into 13 large typical areas, represented by different colors in Figure 8. Eight pathologists (P1-P8 in Table 1) were recruited to rate the trust in the algorithm for classification and generating caption results with the subjective Likert Scale (59). For AI-assisted diagnosis, the baseline average score was 3.88 for the trustworthiness and confidence of AI classification results, while with the visualization results of the AI classification algorithm trained by the CR-PathNarratives dataset, the trust and confidence scores in AI-assisted diagnosis provided with more details raised from 3.88 to 4.63. By providing more auxiliary diagnostic information step by step (reason-layer text description, reason-layer text description, and behavior trajectory thermal map), pathologists’ trust in AI auxiliary diagnosis increased from 4.25 to 4.38. It shows that CR-PathNarratives with decision-to-reason detail benefit the interpretability of AI by doctors.

In conclusion, our dataset can be applied to the basics of classification and captioning scenarios. Experiments show that adding more comprehensive reason information not only achieves better classification gains, identifies detailed features such as cancer stroma, and reduces the false positive rate, but also enhances the trustworthiness and confidence of doctors to understand and collaborate with pathological AI models.

## 6. Conclusion

Pathological diagnosis is the gold standard for tumor diagnosis. The continuous development and progress of AI have brought new possibilities for pathology diagnosis. However, there is a relative lack of datasets in the field of computational pathology. We proposed a data annotation protocol PathNarratives with a hierarchical decision-to-reason data structure and a multimodal annotating process and tool. This data annotation schema focuses on the labeling process of the physician with audit capability, records the behavioral information of the physician, and supports analyzing and discovering the diagnostic ideas and logic of physicians. Based on the protocol we have built the colon-rectal dataset, CR-PathNarratives, which contains 174 H&E-stained WSIs. Each WSI was annotated with decision-to-reason labels and multimodal information on vision, language, voice, and behavioral trajectories. Voice explanations and behavioral trajectories make the data more descriptive. Furthermore, we use the decision-to-reason labels of this dataset to perform classification (adenoma, adenocarcinoma, and normal) experiments, as well as region-level and cluster-level captioning experiments for lesion description. Experiments show that our dataset can be applied to multiscenario algorithmic experiments. Refined annotations facilitate machine learning of more detailed information and reduce the false positive rate of classification. Visualization of comprehensive reasoning details enhances the trustworthiness and confidence of doctors to collaborate with pathological AI models, aiming for better human-AI collaboration.

In the future, we plan to optimize the tools for the annotation process, such as adding automated suggestion hints to speed up the annotation. The WSIs in the datasets are expected to be expanded on 300–800 slides, and then we consider using the proposed annotation model to prepare datasets in other pathological domains. Advanced algorithmic models can be further investigated, e.g., better utilizing behavior tracking as training inputs to optimize the classification results.

## Data availability statement

The original contributions presented in this study are included in the article/Supplementary material, further inquiries can be directed to the corresponding authors.

## Author contributions

HZ, XW, LL, YH, LG, and XS conceived, designed, and coordinated the writing of the whole manuscript. HZ, XW, PH, and WQ contributed to data collection and experiments. HZ, FW, and HC were responsible for software. PH, WQ, FW, HC, JY, XH, and YL revised literature and wrote the different parts of the manuscript. All authors contributed to critically revised and approved the final version of this manuscript.
