# Supplementary material for: PathNarratives: Data annotation for pathological human-AI collaborative diagnosis
Source: Front Med (Lausanne). 2023 Jan 26;9:1070072. doi: 10.3389/fmed.2022.1070072 (PMC9908590; doi:10.3389/fmed.2022.1070072)
Supplement: Supplementary file 1 [file Table_1.docx]

Supplementary Material

# Label terminology

| Classification | Decision-layer annotation | Reason-layer annotation |
| --- | --- | --- |
| adenocarcinoma | Poorly-differentiated adenocarcinoma  Moderately-poorly-differentiated adenocarcinoma  Moderately-differentiated adenocarcinoma  Well-moderately-differentiated adenocarcinoma  Well-differentiated adenocarcinoma | Irregular arrangement of glands  Desmoplastic stroma  Increased layers of epithelial cells  Cribriform  Tumor thrombus  Mucinous differentiation  Mucus pool  Tumor parenchyma  Papillary growth pattern  Micro-papillary growth pattern  Signet ring cell-like  Tubular structure  Villous structure  Necrosis  Chromatin condensation of cells  Mitosis visible  Pathological mitosis  Polar disorder  Stratified or pseudostratified arrangement of nuclei  Vacuolated nuclei  Rod-shaped nuclei  Prominent nucleoli |
|  | Tumor invasion | Invasion into the muscularis mucosae  Invasion into the submucosa  Invasion into the muscularis propria  Invasion into the subserosa  Invasion into the serosa surface  Invasion into the fatty tissue |
|  | Tumor budding | Tumor budding (Grade 1)  Tumor budding (Grade 2)  Tumor budding (Grade 3) |
|  | Vascular invasion |  |
|  | Nerve invasion |  |
| adenoma | High grade adenoma | High-grade intraepithelial neoplasia  Intricately-crowded glands  Chromatin condensation of cells  Mitosis visible  Pathological mitosis  Polar disorder  Stratified or pseudostratified arrangement of nuclei  Vacuolated nuclei  Rod-shaped nuclei  Prominent nucleoli |
|  | Low grade adenoma | Low-grade intraepithelial neoplasia  Serrated structure  Markedly-elongated glands  Glands lack mature differentiation  Largely preserved glandular epithelium and crypt architecture  Markedly reduced cytoplasm  Rod-shaped nuclei  Nucleus stratified or pseudostratified arrangement  Regular arrangement of nuclei  Oval nucleus  Round nucleus |
| normal | normal | Fatty tissue  Smooth muscle  Mucosa layer  Muscularis mucosa  Submucosa  Erythrocyte  Blood vessel  Nerve  Neutrophil  Lymphocyte  Reactive cells  Inflammatory cell"  Infiltration of the lamina propria by inflammatory cells  Inflammatory secretions  Crypts  Mucus  Lymphatic vessel |
